# Effects of Dietary Black Cumin Seed (*Nigella sativa* L.) Meal on Performance, Gut Health, and Meat Quality of Japanese Quail

**DOI:** 10.3390/vetsci13020188

**Published:** 2026-02-13

**Authors:** Kadir Çakır, Hüseyin Çayan

**Affiliations:** 1 Graduate School of Natural and Applied Sciences, Kırşehir Ahi Evran University, 40100 Kırşehir, Türkiye; kadircakir40@gmail.com; 2 Department of Animal Science, Faculty of Agriculture, Kırşehir Ahi Evran University, 40100 Kırşehir, Türkiye

**Keywords:** black cumin seed meal, quail, product quality, MDA, gut health

## Abstract

This study investigated the effects of dietary black cumin seed meal (BCSM) supplementation on growth performance, carcass traits, intestinal histomorphology, cecal microbiota, meat quality, and breast meat malondialdehyde (MDA) levels in Japanese quail. A total of 200 one-week-old quail were randomly allocated to four dietary treatments containing 0, 5, 10, or 20 g/kg BCSM for a 35-day experimental period, with five replicates per treatment. Dietary BCSM supplementation did not significantly affect body weight, body weight gain, feed intake, or conversion ratio (*p* > 0.05). However, carcass weight was significantly increased in birds fed 20 g/kg BCSM (*p* < 0.05), while carcass yield and relative internal organ weight remained unchanged. Intestinal histomorphology was markedly influenced by dietary treatments (*p* < 0.05), with improved villus height and villus height-to-crypt depth ratio in the jejunum and ileum of BCSM-fed birds. In addition, cecal microbiota analysis revealed a dose-dependent increase in *Lactobacillus* spp. (*p* < 0.05), whereas *Escherichia coli* counts were numerically reduced but not statistically affected (*p* > 0.05). Meat quality evaluation showed that BCSM supplementation significantly increased breast meat lightness (L*) and water-holding capacity and reduced post-slaughter pH values (*p* < 0.05). Lipid oxidation, as assessed by malondialdehyde (MDA) concentrations, was observed to be significantly lower in breast meat during refrigerated storage, suggesting a potential improvement in oxidative stability and a possible contribution to extended shelf life (*p* < 0.05). In conclusion, dietary inclusion of black cumin seed meal, particularly at 20 g/kg, positively modulated intestinal health, cecal microbiota composition, and meat oxidative stability without compromising growth performance. Owing to its high nutritional value and rich bioactive compound profile, BCSM can be considered a functional and sustainable feed ingredient for quail nutrition.

## Simple Summary

Black cumin seed (*Nigella sativa* L.) meal is a natural by-product obtained after oil extraction and contains valuable nutrients and bioactive compounds. This study investigated whether adding black cumin seed meal to quail diets could improve production performance, gut health, and meat quality. Feeding quail with diets containing black cumin seed meal did not negatively affect growth or feed intake. However, higher inclusion levels improved feed efficiency, intestinal structure, and beneficial gut bacteria. In addition, meat from quail fed black cumin seed meal showed better water-holding capacity and lower lipid oxidation during storage, indicating improved meat quality and longer shelf life. These results suggest that black cumin seed meal can be safely used as a natural and sustainable feed ingredient in quail production.

## 1. Introduction

The global human population is projected to approach 10 billion by 2050, increasing the demand for safe and high-quality animal protein [1]. Poultry production is well positioned to meet this demand due to its high biological efficiency and short production cycle. Japanese quail (*Coturnix coturnix japonica*) has gained prominence as an alternative meat and egg producer because of its rapid growth, efficient feed utilization and low production cost, while also serving as a suitable model for evaluating novel feed ingredients under intensive production conditions [2].

For many years, antibiotic growth promoters (AGPs) were widely used in poultry diets to enhance growth performance, improve feed conversion and modulate gut health [3]. However, increasing evidence linking AGP use to antimicrobial resistance and residue in animal products has led to strict regulations and bans in many countries. Consequently, the poultry industry has been driven to identify safe and effective alternatives, including organic acids, probiotics, prebiotics, enzymes, and especially phytogenic feed additives derived from medicinal and aromatic plants [4,5,6].

Black cumin (*Nigella sativa* L.), a medicinal and aromatic plant of the *Ranunculaceae* family, is widely used in traditional medicine and as a culinary spice. Its seeds are rich in crude protein, fat, essential fatty acids, minerals, vitamins, and various bioactive compounds such as thymoquinone (27.8–57.0%), p-cymene (7.1–15.5%), carvacrol (5.8–11.6%), t-anethole (0.25–2.3%), 4-terpineol (2.0–6.6%), and longifolene (1.0–8.0%) [6,7,8]. These constituents confer antioxidant, antimicrobial, anti-inflammatory, immunomodulatory and digestive-stimulant properties, and several studies in poultry have reported beneficial effects of black cumin seed or oil on growth performance, feed efficiency, lipid metabolism, intestinal microbiota, immune response and meat quality [6,7,9,10,11].

Black cumin seed meal (BCSM), a by-product of black cumin seed oil extraction, is widely available in regions where black cumin is produced. Although it has traditionally been regarded as a low-value residue, BCSM retains considerable nutritional and functional potential as it contains substantial levels of crude protein, metabolizable energy, dietary fiber, minerals, phenolic compounds, and residual bioactive constituents [12,13,14]. Fathi et al. [12] reported that BCSM includes: 93.18% DM, 31.56% crude protein (CP), 6.41% crude fiber (CF), 11.75% ether extract (EE), 7.12% total ash, 1.16% lysine, 0.56% Dl-Methionine, 0.27% calcium, and 0.35% phosphorus with high nutritive value.

Owing to this favorable nutrient profile, BCSM represents not only a valuable plant-based protein source but also a promising functional feed ingredient. In addition, the presence of bioactive compounds with antimicrobial and antioxidant properties suggests that BCSM may contribute to gut health modulation and could serve as a natural alternative to antibiotic growth promoters in animal nutrition. Accordingly, BCSM has the potential to enhance feed sustainability and reduce reliance on conventional protein sources and synthetic additives [15]. Recent studies in broiler chickens have demonstrated that dietary inclusion of black cumin seed meal can improve growth performance, carcass traits, antioxidant status, immune responses, and caecal microbiota, and may partially or completely replace soybean meal protein without adverse effects on performance or meat quality [12,16,17,18,19].

Despite these promising results in broilers, information on the use of black cumin seed meal in quail nutrition is very limited, and available data mainly concern whole seeds or essential oils rather than the meal. In addition, there is a lack of comprehensive studies evaluating not only growth performance and carcass characteristics but also gut health and meat quality responses to graded inclusion levels of black cumin seed meal in quail diets. Therefore, the present study was conducted to investigate the effects of dietary inclusion of different levels of black cumin seed meal in Japanese quail diets on growth performance, intestinal morphology, and microbiota and meat quality traits.

## 2. Materials and Methods

### 2.1. Animals and Experimental Design

A 35-day feeding trial was conducted using a total of 200 unsexed, 7-day-old Japanese quail (*Coturnix coturnix japonica*) chicks with an average initial body weight of 20.63 g. The birds were randomly assigned to four dietary treatments containing 0 (control), 5, 10, and 20 g/kg black cumin seed meal (BCSM). Each treatment consisted of five replicates with 10 birds per replicate.

Birds were fed for a period of five weeks in accordance with NRC recommendations [20], using a commercially sourced broiler grower diet. The basal ration provided 22% crude protein and 3100 kcal/kg metabolizable energy and contained 0.92 g/kg calcium and 0.47 g/kg phosphorus. This diet was offered either as the unsupplemented control or supplemented with black cumin seed meal.

The black cumin seed meal used in the study was obtained from the Aegean Agricultural Research Institute as a by-product of the cold-press oil extraction process of the Çameli cultivar. The seed meal was ground to pass through a 1 mm sieve and incorporated into the basal diet at the designated levels, ensuring homogeneous mixing. Proximate analysis of black cumin seed meal was conducted at the Central Research and Application Laboratory, Kırşehir Ahi Evran University. The dry matter-based composition was as follows: dry matter, 95.0%; crude protein, 36.05%; ether extract, 5.20%; crude ash, 6.0%; starch, 3.04%; and crude fiber, 13.0%.

The birds were reared in an environmentally controlled room using a four-level battery cage system arranged in five blocks, comprising a total of 20 stainless-steel cages (92 × 33 × 45 cm). Feed and drinking water were provided freely throughout the trial. A near-continuous lighting program (23 h light:1 h dark) was applied. To reduce potential cage-location effects, birds were evenly distributed between the upper and lower tiers. Ambient temperature was set at 33 ± 1 °C during the first week and then progressively decreased to 24 ± 1 °C for the remainder of the experiment. Relative humidity was maintained at levels exceeding 60% throughout the study period.

### 2.2. Determination of Performance Parameters

The experiment involved body weight monitoring on days 7, 14, 21, 28, 35 and 42. Feed intake (FI) was estimated by measuring the feed offered and subtracting the leftovers. Feed conversion ratio (FCR) was calculated using the equation FCR = FI/BWG.

### 2.3. Slaughtering Process and Carcass Measurements

On day 42, corresponding to the end of the experimental period, all birds were individually weighed to obtain final body weight data. From each treatment group, two birds (one female and one male) with live weight close to the group average were selected from each replicate, for a total of 40 birds (10 birds per group), which were then slaughtered and subjected to subsequent analysis. Following slaughter, carcass yield was calculated based on a ready-to-cook carcass (fully eviscerated) and the hot carcass yield was used. The length of the digestive tract was measured using a measuring tape, and the weights of the heart, gizzard (with the lining removed), and liver were recorded using a precision balance with an accuracy of 0.01 g.

The pH measurements were taken from the breast muscles of each cleaned carcass after slaughter and 24 h after slaughter. The pH of the breast muscle was recorded at three sites on the left side of the carcass using a Testo 205 digital pH meter (Testo SE & Co. KGaA, Titisee-Neustadt, Germany) with a solid-type electrode.

Color measurements of the breast muscle were obtained from the skinless surface using a Minolta CR-410 Chroma Meter (Konica Minolta, Inc., Tokyo, Japan), calibrated with a white reference plate. Readings were taken at three different locations per sample and averaged for analysis.

Water-holding capacity (WHC) was analyzed 24 h after slaughter using the pressing method [21]. One gram minced meat samples were placed between Whatman No. 1 filter papers (Cytiva, Maidstone, UK) and pressed with a 2.25 kg weight for 5 min. The obtained value was expressed as % hygroscopicity.

Thawing loss was determined according to the method described by Mortensen et al. [22]. Breast muscle samples were frozen at −20 °C and subsequently thawed at +4 °C for 24 h. Thawing loss (%) was calculated as the difference between the sample weights before freezing and after thawing.

Drip loss was assessed following the procedure described by Bond and Warner [23]. Approximately 5–10 g breast meat samples were vacuum-packed and stored at +4 °C. After 72 h and 148 h of storage, samples were removed from the bags, gently blotted dry, and weighed. Drip loss (%) was calculated as the percentage weight difference between the initial and final measurements.

Cooking loss was measured following Nuwan et al. [24]. Approximately 5 g breast samples were vacuum-sealed and cooked in a water bath at 80 °C for 40 min, then cooled to room temperature. After blotting and reweighing, cooking loss (%) was calculated from the weight difference before and after cooking.

### 2.4. Evaluation of Small Intestinal Histomorphology

On the 42nd day of the study, jejunal and ileal samples from slaughtered animals in each treatment group were placed in 10% formaldehyde. For histological analysis, paraffin blocks were prepared, the samples were cut to a thickness of 5 μm, and the tissues were adhered to the slide. The tissues on the slide were freed from paraffin by passing through xylene, which was then passed through alcohol, and xylene was removed from the tissues. The cleaned tissue samples were stained with hematoxylin and eosin dye and photographed using an AxioCam ERc 5 s 5 megapixel digital microscope camera (ZEISS Primo Star, Jena, Germany). Villi length and crypt depth measurements from photographs of each treatment group and sample were made using the ZEN 2012 SP2 image processing and analysis software, blue edition (Carl Zeiss Microscopy GmbH, Jena, Germany) [25,26].

### 2.5. Determination of Meat Shelf Life

Lipid oxidation of meat samples stored at +4 °C was determined on days 1 and 3 post-slaughter. Lipid peroxidation was assessed using a modified 2-thiobarbituric acid (TBA) method as described by Ke et al. [27]. Results were expressed as thiobarbituric acid reactive substances (TBARSs), reported as mg malondialdehyde (MDA) per kg of sample. The principle of the method is based on the formation of a red-colored complex resulting from the reaction between malondialdehyde, a secondary lipid oxidation product, and thiobarbituric acid following heat treatment. For analysis, 10 g of each meat sample was homogenized with distilled water using an Ultra-Turrax homogenizer (IKA T18, Staufen, Germany). The homogenate was transferred to a Kjeldahl flask, acidified with 2.5 mL of 4 N HCl (Merck, Darmstadt, Germany), and a small amount of paraffin was added to minimize foaming. The mixture was then distilled, and 5 mL of the distillate was collected. Subsequently, 5 mL of thiobarbituric acid reagent (Merck, Germany) was added to the distillate, and the mixture was incubated in a water bath for 30 min. After cooling, the absorbance of the resulting solution, along with a blank, was measured spectrophotometrically at 538 nm. The absorbance values were multiplied by a factor of 7.8 to calculate TBARS concentration. Final results were expressed as mg MDA/kg of meat sample [28].

### 2.6. Analysis of Cecal Microbial Populations

Cecal digesta (1 g) was collected from two birds per replicate (40 samples; 10 per treatment). Each sample was suspended in 9 mL peptone water, vortexed, and serially diluted (10-fold). *Lactobacillus* spp., and *E. coli* were enumerated on MRS (Merck 1.10660), and EMB (Merck 101347) agars, incubated for 72 h at 37 °C, and 37 °C, respectively. Results were expressed as log_10_ CFU g^−1^ of cecal digesta [29].

### 2.7. Statistical Analysis

The data obtained from the experiment were analyzed using a one-way analysis of variance (ANOVA) based on a completely randomized design. Differences among the group means were evaluated using Duncan’s multiple range test, and the results were recorded accordingly. All statistical analyses were performed using the SPSS 21.0 for Windows Evaluation Version statistical software package.

## 3. Results

### 3.1. Performance Parameters

The effects of increasing dietary levels of black cumin seed meal (BCSM) on growth performance of Japanese quail are summarized in Table 1.

Body weight (BW) at 7, 21, and 42 days of age was not significantly affected by dietary BCSM supplementation (*p* > 0.05). Similarly, daily body weight gain (DBWG) during the periods of 7–21, 21–42, and 7–42 days did not differ among treatments (*p* > 0.05).

Feed intake (FI) was not significantly influenced by dietary treatments during any experimental period (*p* > 0.05), although a numerical reduction in FI was observed as the dietary BCSM level increased.

Feed conversion ratio (FCR) exhibited a progressive numerical improvement with increasing dietary BCSM inclusion across all periods. While FCR during the 7–21- and 21–42-day periods were not statistically different among treatments (*p* > 0.05), overall FCR for the entire experimental period (7–42 days) was significantly affected by dietary BCSM levels (*p* = 0.025). Birds fed 20 g/kg BCSM showed the lowest FCR compared with the control and 5 g/kg groups.

### 3.2. Carcass and Visceral Organs

The effects of dietary black cumin seed meal (BCSM) supplementation on slaughter characteristics, carcass traits and visceral organ weights of Japanese quail are presented in Table 2.

Slaughter weight was not significantly affected by dietary BCSM inclusion (*p* > 0.05), although birds fed 20 g/kg BCSM exhibited a numerically higher slaughter weight compared with the other groups. Similarly, carcass yield (%) did not differ among treatments (*p* > 0.05), despite a numerical increase observed in the 20 g/kg BCSM group.

In contrast, carcass weight was significantly influenced by dietary treatment (*p* = 0.029). Birds receiving 20 g/kg BCSM showed a significantly higher carcass weight compared with the control, 5 g/kg, and 10 g/kg groups, indicating an improvement in carcass deposition at the highest inclusion level.

Relative weights of the heart, liver, gizzard and edible internal organs (EIOs), expressed as g/100 g body weight, were not affected by dietary BCSM supplementation (*p* > 0.05). Gastrointestinal length (GL) also did not differ statistically among treatments (*p* > 0.05); however, a numerical increase was observed in birds fed 20 g/kg BCSM.

### 3.3. Meat Quality and Shelf Life

The effects of dietary black cumin seed meal (BCSM) supplementation on meat quality traits and shelf-life parameters of Japanese quail are presented in Table 3.

According to Table 3, breast meat pH measured immediately after slaughter was significantly affected by dietary BCSM inclusion (*p* = 0.018). Birds fed 5 g/kg BCSM exhibited a lower pH-0 value compared with the control group, whereas the 10 and 20 g/kg groups showed no differentiation between control and BCSM-5 groups. In contrast, breast meat pH measured at 24 h postmortem (pH-24) did not differ among dietary treatments (*p* > 0.05).

Dietary BCSM supplementation significantly influenced breast meat lightness (L* value) (*p* = 0.001). The highest L* value was observed in birds fed 20 g/kg BCSM, indicating a lighter breast meat color compared with the other groups.

Water-holding capacity (WHC) was significantly improved by increasing dietary BCSM levels (*p* = 0.003). Birds fed 20 g/kg BCSM exhibited the highest WHC, indicating enhanced water retention capacity of breast meat.

Thawing loss, cooking loss, and drip loss measured at 3 and 7 days of refrigerated storage were not significantly affected by dietary BCSM supplementation (*p* > 0.05), although numerically higher drip and thawing losses were observed in the BCSM-supplemented groups.

Dietary BCSM supplementation markedly affected lipid oxidation levels in breast meat, as indicated by malondialdehyde (MDA) concentrations. MDA values measured on day 1 of storage (MDA-1) were significantly reduced in all BCSM-supplemented groups compared with the control (*p* < 0.001), with the lowest value observed in the 20 g/kg BCSM group. Similarly, MDA values measured on day 3 (MDA-3) were significantly lower in BCSM-fed birds than in the control group (*p* = 0.004).

### 3.4. Small Intestinal Histomorphology

**Figure 1:**
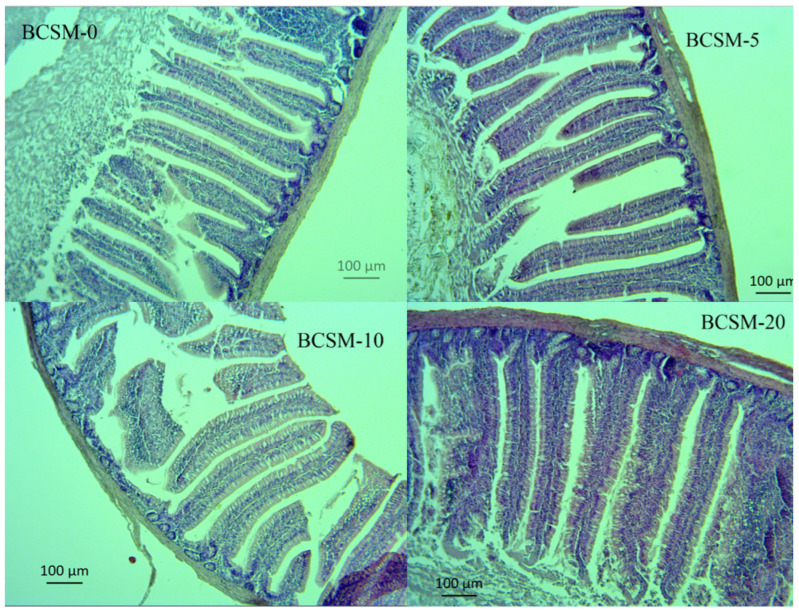
Cross-sections of the quail jejunum from those fed with black cumin seed meal (BCSM scale bar 200 µm, 10× magnification). Images of jejunum villi of quails from: (BCSM-0) control group, (BCSM-5) fed with 5 g/kg black cumin seed meal group, (BCSM-10) fed with 10 g/kg black cumin seed meal group, and (BCSM-20) fed with 20 g/kg black cumin seed meal group.

**Figure 2:**
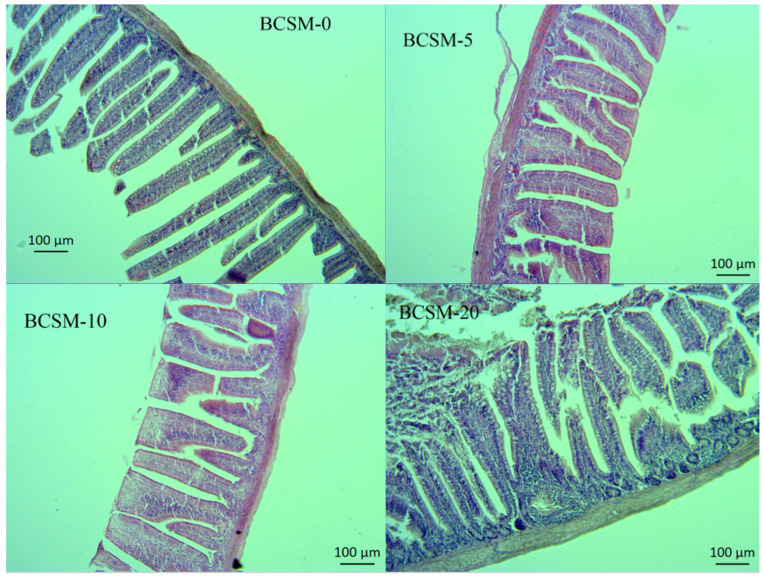
Cross-sections of the quail ileum from those fed with black cumin seed meal (BCSM scale bar 200 µm, 10× magnification). Images of jejunum villi of quails from: (BCSM-0) control group, (BCSM-5) fed with 5 g/kg black cumin seed meal group, (BCSM-10) fed with 10 g/kg black cumin seed meal group, and (BCSM-20) fed with 20 g/kg black cumin seed meal group.

The effects of dietary black cumin seed meal (BCSM) supplementation on jejunal and ileal histomorphological parameters of Japanese quail are presented in Table 4, and figures are given in Figure 1 and Figure 2.

Jejunal villus length was significantly affected by dietary BCSM inclusion (*p* < 0.05). Birds fed 5 g/kg BCSM exhibited the greatest villus height, followed by the 10 and 20 g/kg groups, whereas the control group showed the lowest villus length. Jejunal crypt depth was also significantly influenced by dietary treatment (*p* = 0.004), with the deepest crypts observed in the control group and reduced crypt depth in BCSM-supplemented groups, particularly at 5 g/kg.

The villus height-to-crypt depth ratio (VH:CD) in the jejunum differed significantly among treatments (*p* < 0.001). BCSM-supplemented groups showed a markedly higher VH:CD ratio compared with the control group, indicating improved intestinal absorptive capacity. Although the highest ratio was observed in the 5 g/kg group, the 10 and 20 g/kg groups also maintained significantly higher values than the control.

Ileal villus length increased significantly with increasing dietary BCSM levels (*p* < 0.001). The longest villi were observed in birds fed 20 g/kg BCSM, while the control and 5 g/kg groups exhibited the shortest villi. Ileal crypt depth was also affected by dietary treatment (*p* = 0.015), with deeper crypts observed in the 10 and 20 g/kg groups compared with the control.

The villus height-to-crypt depth ratio in the ileum showed a significant treatment effect (*p* < 0.001). Birds receiving 20 g/kg BCSM exhibited the highest VH:CD ratio, indicating a marked improvement in ileal mucosal development and functional capacity.

### 3.5. Cecal Microbiota

The effects of black cumin seed meal supplementation into quails on cecal microbiota are presented in Table 5. Dietary supplementation of black cumin seed meal (BCSM) significantly affected the cecal *Lactobacillus* spp. population in Japanese quail (*p* < 0.001). Birds fed BCSM-supplemented diets exhibited higher *Lactobacillus* spp. counts compared with the control group, with the highest values observed in the 20 g/kg BCSM group. In contrast, *Escherichia coli* counts were not significantly influenced by dietary treatments (*p* > 0.05), although numerically lower values were recorded in quails receiving BCSM compared with the control.

## 4. Discussion

The results demonstrated that dietary supplementation of BCSM did not significantly affect BW, DBWG or FI of Japanese quail throughout the experimental period. These findings indicate that BCSM can be included in quail diets up to 20 g/kg without adverse effects on growth performance, suggesting good palatability and nutrient utilization. Although most growth performance parameters were not statistically different among treatments, birds fed higher levels of BCSM, particularly 20 g/kg, showed numerically greater final body weight and daily weight gain compared with the control group. Such numerical improvements, despite not reaching statistical significance, may be biologically relevant and are commonly reported in studies evaluating phytogenic feed additives, where responses often depend on dose, duration, animal age, and basal diet composition [30,31]. The numerical increases observed in body weight and body weight gain with increasing dietary levels of black cumin seed meal (BCSM) may be attributed to the nutritional and bioactive composition of this by-product. Albakry et al. [32] have reported that BCSM is relatively rich in unsaturated fatty acids, particularly linoleic and linolenic acids, which may enhance energy utilization and support growth performance. In addition, secondary metabolites present in black cumin seed meal, such as p-cymene, thymoquinone and di-thymoquinone, have been reported to stimulate digestive enzyme activity, thereby improving nutrient digestibility and absorption [33,34]. Information regarding the use of black cumin seed meals in quail diets is limited, with only one previous study directly evaluating its inclusion. Abd El-Hack et al. [35] reported a dose-dependent increase in the body weight of Japanese quail fed increasing levels of black cumin seed meal, which is consistent with the numerical trends observed in the present study. Moreover, studies investigating the use of whole black cumin seeds or their derivatives in quail nutrition have similarly reported positive effects on body weight and growth performance [7,11,36]. In broiler chicks, several studies have demonstrated improvements in body weight when black cumin seed meal was included in the diet [12,15,16,17,37,38], whereas others reported no significant effects on growth performance [39,40]. These inconsistencies among studies may be related to differences in inclusion level, processing method, basal diet composition, animal species and age, as well as experimental duration.

In the present study, dietary inclusion of black cumin seed meal (BCSM) did not significantly affect feed intake of Japanese quail throughout the experimental period. This finding indicates that supplementation with BCSM at levels up to 20 g/kg does not compromise feed palatability or voluntary feed consumption. The absence of a significant effect on feed intake is in agreement with previous studies reporting no alterations in feed intake when black cumin seeds or by-products were included in quail diets [11,16,41]. Similarly, studies conducted in broiler chickens have demonstrated that dietary inclusion of black cumin seed or black cumin seed meal did not significantly influence feed intake [18,40]. Collectively, these findings support the notion that black cumin-based ingredients can be incorporated into poultry diets without adversely affecting feed acceptability. Conversely, some studies have reported a reduction in feed intake with increasing inclusion levels of black cumin seed or seed meal. Abd El-Hack et al. [35] and Shokrollahi and Sharifi [36] observed decreased feed intake in quail fed higher dietary levels of black cumin seed, while El-Kashef [17] reported a negative effect on feed intake in broiler chickens at relatively high inclusion rates (up to 9%). Such reductions in feed intake at elevated doses may be associated with the intense aroma, bitter taste, or increased dietary fiber content of black cumin seed products, which may limit voluntary feed consumption when inclusion levels exceed a certain threshold [42]. In contrast to these findings, Edmonds et al. [43] and Sogut et al. [44] reported improved feed intake in broiler chickens fed diets supplemented with black cumin seeds compared with control diets. These inconsistencies among studies may be attributed to differences in the form of black cumin used (whole seed vs. meal), inclusion level, processing method, basal diet composition, animal species, age, and duration of the experimental period.

Notably, the overall feed conversion ratio (FCR) during the 7–42 d experimental period was significantly improved in birds fed 20 g/kg BCSM. This enhancement in feed efficiency may be attributed to the presence of residual bioactive compounds in BCSM, such as thymoquinone and various phenolic constituents, which are known to exert antimicrobial and antioxidant effects and may thereby improve gut functionality and nutrient absorption. The observed improvement in FCR is further supported by the concomitant enhancements in intestinal histomorphology and cecal microbiota recorded in the present study. In particular, increased villus height and villus height-to-crypt depth ratio, especially in the ileum, indicate an expansion of the intestinal absorptive surface area, which may facilitate more efficient nutrient uptake. Moreover, the increased abundance of beneficial *Lactobacillus* spp. in the cecum may have contributed to improved gut health, microbial balance, and digestive efficiency, ultimately resulting in better feed utilization. Consistent with these findings, previous studies in broiler chickens have reported improvements in feed efficiency without marked changes in body weight gain following dietary supplementation with black cumin seed meal or oil, with enhanced intestinal morphology and modulation of gut microbiota proposed as key mechanisms underlying improved nutrient utilization [12,16,35,45]. The present results extend these observations to Japanese quail and suggest that BCSM supplementation may primarily enhance feed efficiency rather than directly stimulating growth rate.

In the present study, dietary supplementation of black cumin seed meal (BCSM) did not significantly affect slaughter weight or carcass yield of Japanese quail, whereas carcass weight was significantly increased in birds fed 20 g/kg BCSM. These findings indicate that BCSM inclusion at higher levels may enhance carcass deposition without adversely affecting overall growth or dressing percentage. The lack of a significant effect on slaughter weight, despite the numerical increase observed in the highest BCSM group, suggests that the improvement in carcass weight may be related to more efficient tissue deposition rather than an increase in live body weight. This interpretation is further supported by the absence of significant changes in relative organ weight, indicating that BCSM supplementation did not induce disproportionate development of visceral organs. The observed increase in carcass weight at 20 g/kg BCSM is consistent with previous reports demonstrating positive effects of black cumin seed or seed meal on carcass traits in poultry. Jahan et al. [16] reported increased carcass weight and improved economic efficiency in broilers fed diets containing black cumin seed meal, while El-Kashef [17] observed favorable effects on carcass characteristics at moderate inclusion levels. Similar improvements in carcass-related parameters have also been reported in studies evaluating black cumin seed products in poultry diets [12,38]. In contrast, several studies have reported no significant effects of black cumin seeds or by-products on carcass yield or slaughter characteristics [40,46], suggesting that the response may depend on factors such as inclusion level, form of the additive (whole seed, oil, or meal), processing method, and animal species. In quail specifically, limited data are available; however, Abd El-Hack et al. [35] reported numerical increases in carcass-related traits with increasing levels of black cumin seed meal, which aligns with the trend observed in the present study.

The effects of dietary black cumin seed meal (BCSM) supplementation on meat quality traits and shelf life of Japanese quail were evaluated based on breast meat characteristics. The results indicate that dietary BCSM influenced selected meat quality parameters, particularly breast meat lightness (L*), early postmortem pH, water-holding capacity (WHC), and lipid oxidation, while other physical quality traits remained largely unaffected. In the present study, breast meat lightness (L*) was significantly affected by dietary treatment, with the highest L* values observed in birds fed 20 g/kg BCSM. This finding suggests that higher inclusion levels of BCSM may result in a lighter breast meat appearance, which is generally perceived as a desirable quality attribute by consumers. Similar increases in breast meat lightness have been reported in quail fed diets supplemented with black cumin seed at different inclusion levels, supporting the consistency of the present findings [11].

Meat pH is a critical determinant of meat quality, as it directly influences color, water-holding capacity, texture and shelf life [47]. In this study, dietary BCSM supplementation significantly affected breast meat pH measured immediately after slaughter, whereas pH measured at 24 h postmortem was not influenced by dietary treatments. The reduction in early postmortem pH observed in BCSM-supplemented groups may be associated with the bioactive constituents of BCSM, which possess antioxidant, antimicrobial, and metabolic-modulating properties. These compounds may limit lipid oxidation and the formation of alkaline by-products during early postmortem metabolism, thereby contributing to lower pH values. In agreement with the present results, Asghar et al. [11] reported reduced pH values in quail meat with increasing dietary inclusion of black cumin seed at both slaughter and during subsequent storage periods. In contrast, Fathi et al. [12] observed increased pH values in broiler meat with higher levels of black cumin seed meal, suggesting that species differences, inclusion level, and the form of black cumin used may influence postmortem pH responses.

Among the evaluated meat quality parameters, WHC was the only trait that showed a significant and dose-dependent improvement with increasing dietary BCSM levels. Water-holding capacity is a key indicator of meat quality, reflecting the ability of muscle tissue to retain water after slaughter, and is closely associated with juiciness and processing yield. Szmańko et al. [48] reported that increased WHC, decreased muscle juice (water) loss during storage, minimizing economic losses. The observed increase in WHC may be attributed to the antioxidant metabolites present in BCSM, which can reduce protein and lipid oxidation, preserve cellular integrity, and maintain the functionality of myofibrillar proteins responsible for water binding. A positive relationship between meat pH and WHC has been widely reported, and dietary antioxidants have been shown to modulate proteolytic processes during postmortem aging, thereby improving WHC [47,49]. Accordingly, the improved WHC observed in birds fed 20 g/kg BCSM suggests an enhancement of the antioxidative defense system in muscle tissue. Other physical quality traits, including thawing loss, cooking loss, and drip loss measured at days 3 and 7 of refrigerated storage, were not significantly affected by dietary treatments. Nevertheless, a numerical improvement in these parameters was observed in BCSM-supplemented groups, indicating a potential tendency toward improved moisture retention, although these differences were not statistically significant under the conditions of the present study.

Lipid oxidation is one of the most important factors limiting meat shelf life, as it leads to rancidity and quality deterioration. In the present study, malondialdehyde (MDA) concentrations measured after 1 and 3 days of refrigerated storage were significantly reduced in breast meat from birds fed BCSM-supplemented diets. The lowest MDA values were consistently observed in the 20 g/kg BCSM group, indicating a strong dose-dependent antioxidant effect. The antioxidant activity of black cumin is largely attributed to its rich content of phenolic compounds and volatile oil constituents, particularly thymoquinone, which accounts for approximately 60–80% of the essential oil fraction, along with other compounds such as anethole, 4-terpineol and carvacrol. These bioactive components exhibit strong free-radical scavenging activity and effectively inhibit lipid peroxidation [9,50,51,52]. The present findings are consistent with previous studies reporting reduced MDA levels in quail meat following dietary supplementation with black cumin seed [11] and in broiler meat supplemented with black cumin seed meal [12]. Collectively, these results indicate that BCSM can effectively delay lipid oxidation and extend the shelf life of quail meat.

In the present study, dietary supplementation of black cumin seed meal (BCSM) significantly influenced the histomorphological characteristics of the jejunum and ileum in Japanese quail, indicating a clear effect of dietary treatment on intestinal structure and gut health. Jejunal villus height, crypt depth, and villus height-to-crypt depth ratio (VH:CD) were significantly improved in BCSM-supplemented groups compared with the control. The most pronounced improvements in jejunal morphology were observed in birds fed 5 g/kg BCSM, suggesting that lower inclusion levels may be sufficient to stimulate jejunal mucosal development. Increased villus height and VH:CD ratio are widely accepted indicators of enhanced absorptive capacity and reduced epithelial turnover, which are closely associated with improved nutrient digestion and absorption. In contrast to the jejunum, ileal histomorphological parameters exhibited a dose-dependent response, with the highest villus height, crypt depth and VH:CD ratio observed in birds fed 20 g/kg BCSM. This finding suggests that higher inclusion levels of BCSM may exert a more pronounced effect on the distal segments of the small intestine, potentially reflecting region-specific sensitivity to dietary bioactive compounds.

Intestinal villi and crypts play a fundamental role in maintaining gut integrity and functionality. Villi are the primary sites for nutrient absorption, while crypts are responsible for epithelial cell proliferation and renewal. An increase in villus height and VH:CD ratio is associated with an expanded absorptive surface area and more efficient nutrient utilization, which may ultimately translate into improved growth performance [53]. Accordingly, the numerical improvements observed in performance parameters in the present study may be partially attributed to the observed enhancements in intestinal morphology. The beneficial effects of BCSM on intestinal histomorphology observed in this study are consistent with previous findings in poultry. Improvements in villus height and VH:CD ratio have been reported in broiler chickens fed diets supplemented with black cumin seed meal [38,40], whole black cumin seeds [54], and black cumin oil [55]. These studies collectively suggest that black cumin-derived products can positively modulate intestinal architecture across different poultry species. The mechanisms underlying these effects are likely related to the bioactive constituents of black cumin, including phenolic compounds and thymoquinone, which possess antimicrobial, antioxidant and anti-inflammatory properties. These compounds may contribute to improved gut health by modulating intestinal microbiota, reducing oxidative stress, and stabilizing epithelial cell membranes, thereby promoting villus development and reducing excessive crypt hyperplasia. In line with this interpretation, Abdollahi et al. [54] suggested that dietary black cumin supplementation may enhance nutrient absorption efficiency while reducing intestinal epithelial turnover.

At the end of the 42-day experimental period, cecal microbiological analysis revealed that dietary supplementation of black cumin seed meal (BCSM) significantly affected the population of lactic acid bacteria, whereas *Escherichia coli* counts were not significantly influenced by dietary treatments. A clear dose-dependent increase in *Lactobacillus* spp. was observed with increasing dietary BCSM levels, with the highest counts recorded in birds fed 20 g/kg BCSM. Although *E. coli* populations were not statistically different among treatments, numerically lower values were observed in BCSM-supplemented groups compared with the control.

The observed increase in *Lactobacillus* spp. populations may be attributed to the bioactive composition of black cumin seed meal. Black cumin contains essential oil components such as thymoquinone, carvacrol and 4-terpineol, which exhibit selective antimicrobial activity against pathogenic bacteria, particularly Gram-negative organisms such as *E. coli*, while exerting minimal inhibitory effects on beneficial lactic acid bacteria [9]. In addition, the polyphenols, flavonoids and dietary fibers present in black cumin seed meal may serve as substrates that promote the proliferation of beneficial microbiota in the cecum. An increased abundance of lactic acid bacteria is associated with a reduction in intestinal pH due to lactic acid production, thereby creating an unfavorable environment for pathogenic microorganisms. This shift in microbial balance toward beneficial bacteria is considered an important indicator of improved gut health and may contribute to enhanced digestive efficiency and nutrient utilization. Consequently, the numerical improvements observed in growth performance and feed conversion efficiency in the present study may be partially explained by the favorable modulation of cecal microbiota.

The present findings are in agreement with previous studies reporting beneficial effects of black cumin-derived products on intestinal microbiota. Adegbeye et al. [38] demonstrated that dietary inclusion of black cumin seed meal increased lactic acid bacteria populations while suppressing *E. coli* in the cecum of broiler chickens. Similarly, Abdollahi et al. [54] reported a significant increase in ileal lactic acid bacteria and a concomitant reduction in coliform counts in broilers fed diets supplemented with black cumin seeds. In contrast, Laudadio et al. [56] reported no significant effects of black cumin seed supplementation on cecal microbiota in broiler chickens. Such discrepancies among studies may be attributed to differences in the form of black cumin used (whole seeds vs. meal), inclusion level, basal diet composition, bird species and age, and analytical methodologies used for microbiota assessment.

## 5. Conclusions

Inclusion of 20 g/kg BCSM develops feed conversion ratio, intestinal histomorphology, cecal *Lactobacillus* spp. abundance, and meat oxidative stability during refrigerated storage. Collectively, these results suggest that BCSM may serve as an effective alternative phytobiotic feed additive, promoting gut health and nutrient utilization while valorizing an agro-industrial by-product. The results of this study have revealed the necessity of determining the effects of BCSM under commercial conditions, different housing systems, disease challenge conditions, or other stress factors.

## Figures and Tables

**Table 1:** Growth performance of Japanese quail (7–42 d) fed diets supplemented with black cumin seed meal.

Parameters	BCSM Level (g/kg)				SEM	*p*-Value
	0	5	10	20		
BW (g)						
7 d	21.68	21.64	21.60	21.60	0.016	0.261
21 d	86.09	88.14	87.84	90.88	0.753	0.155
42 d	187.75	187.52	189.33	198.97	2.101	0.167
DBWG (g/d)						
7–21	4.60	4.75	4.73	4.95	0.054	0.147
21–42	4.84	4.73	4.83	5.15	0.076	0.256
7–42	4.74	4.74	4.79	5.07	0.060	0.165
FI (g)						
7–21	13.69	13.50	13.08	13.36	0.121	0.355
21–42	27.05	25.98	25.68	25.58	0.274	0.212
7–42	21.71	20.99	20.64	20.69	0.183	0.138
FCR (g feed/g gain)						
7–21	2.98	2.85	2.77	2.70	0.041	0.079
21–42	5.59	5.50	5.32	5.01	0.088	0.083
7–42	4.58 ^a^	4.43 ^a^	4.31 ^ab^	4.10 ^b^	0.061	0.025

Results are expressed as averages derived from five replicate units, each represented by two birds; ^a,b^ different superscript letters indicate significant differences among means (*p* < 0.05); SEM: standard error of means; DBWG: daily body weight gain; FI: feed intake; FCR: feed conversion ratio (feed intake/weight gain).

**Table 2:** Visceral organ and carcass traits of quail fed a diet containing black cumin seed meal (g/100 g BW).

Parameter	BCSM Level (g/kg)				SEM	*p*-Value
	0	5	10	20		
Slaughter weight (g)	189.70	184.70	187.00	197.40	2.049	0.139
Carcass weight (g)	126.25 ^b^	123.76 ^b^	124.41 ^b^	136.40 ^a^	1.722	0.029
Carcass yield (%)	66.59	66.10	67.02	68.98	0.649	0.432
Heart weight	1.01	0.99	1.01	1.02	0.018	0.904
Liver weight	2.61	2.09	2.07	2.17	0.104	0.232
Gizzard weight	1.98	2.12	2.20	2.07	0.053	0.535
EIO weight ^1^	5.60	5.19	5.27	5.27	0.128	0.689
GL (cm)	59.1	56.7	57.2	61.7	0.752	0.073

Results are expressed as averages derived from five replicate units, each represented by two birds; ^a,b^ different superscript letters indicate significant differences among means (*p* < 0.05); ^1^ the weights of the heart, liver, and gizzard; SEM: standard error of means; EIO: edible inner organs; BW: body weight; GL: gut length.

**Table 3:** Breast meat quality and shelf-life parameters of quails fed diets supplemented with black cumin seed meal.

Parameters	BCSM Level (g/kg)				SEM	*p*-Value
	0	5	10	20		
pH-0	5.69 ^a^	5.03 ^b^	5.45 ^ab^	5.40 ^ab^	0.075	0.018
pH-24	5.79	5.88	6.00	5.93	0.033	0.181
L*	41.18 ^b^	40.04 ^b^	41.11 ^b^	42.94 ^a^	0.258	0.001
a*	3.34	3.75	3.79	3.61	0.132	0.632
b*	6.97	7.36	7.10	7.65	0.111	0.145
Water-holding capacity (%)	10.53 ^b^	11.08 ^b^	13.05 ^b^	17.80 ^a^	0.894	0.003
Thawing loss (%)	1.65	2.62	1.98	3.04	0.260	0.257
Cooking loss (%)	27.87	28.50	25.53	27.85	0.483	0.130
Drip loss 3 (%)	1.98	2.39	2.01	2.48	0.211	0.809
Drip loss 7 (%)	5.97	6.95	6.53	6.78	0.225	0.489
MDA-1	0.35 ^a^	0.28 ^b^	0.27 ^b^	0.19 ^c^	0.013	0.000
MDA-3	0.40 ^a^	0.29 ^b^	0.28 ^b^	0.23 ^b^	0.019	0.004

Results are expressed as averages derived from five replicate units, each represented by two birds; ^a–c^ different superscript letters indicate significant differences among means (*p* < 0.05); SEM: standard error of means; L*: lightness; a*: redness; b*: yellowness; drip loss 3/7: drip loss (%) measured after 3/7 days of refrigerated storage at 4 °C; MDA-1/3: (mg/kg) malondialdehyde concentration measured on day 1/3 of refrigerated storage (+4 °C).

**Table 4:** Intestinal histomorphology of quails with dietary black cumin seed meal.

Parameters	BCSM Level (g/kg)				SEM	*p*-Value
	0	5	10	20		
Jejunum						
VL (μm)	497.08 ^c^	590.31 ^a^	545.79 ^b^	546.59 ^b^	7.363	0.000
CD (μm)	40.73 ^a^	32.85 ^b^	37.04 ^ab^	37.02 ^ab^	0.779	0.004
VL/CD	11.38 ^c^	18.48 ^a^	15.44 ^b^	15.58 ^b^	0.406	0.000
İleum						
VL (μm)	351.85 ^c^	347.33 ^c^	386.40 ^b^	465.96 ^a^	5.87	0.000
CD (μm)	30.00 ^b^	31.34 ^ab^	33.50 ^a^	34.32 ^a^	0.535	0.015
VL/CD	12.34 ^b^	11.32 ^b^	11.84 ^b^	14.18 ^a^	0.244	0.000

Results are expressed as averages derived from five replicate units, each represented by two birds; ^a–c^ different superscript letters indicate significant differences among means (*p* < 0.05); SEM: standard error of means; VL: villi length; CD: crypt depth; VL/CD: villi length/crypt depth.

**Table 5:** Cecum microbiota of quails with dietary supplementation black cumin seed meal (CFU g^−1^).

Parameters	BCSM Level (g/kg)				SEM	*p*-Values
	0	5	10	20		
*Lactobacillus* spp.	6.78 ^c^	7.58 ^ab^	7.27 ^b^	7.95 ^a^	0.105	0.000
*Escherichia coli*	7.70	7.62	7.42	7.56	0.094	0.803

Results are expressed as averages derived from five replicate units, each represented by two birds; ^a–c^ different superscript letters indicate significant differences among means (*p* < 0.05); SEM: standard error of means; CFU: colony-forming units.

## Data Availability

The original contributions presented in this study are included in the article. Further inquiries can be directed to the corresponding author.

## Abbreviations

The following abbreviations are used in this manuscript:

a*	Redness
AGP	Antibiotic growth promoter
b*	Yellowness
BCSM	Black cumin seed meal
BWG	Body weight gain
CD	Crypt depth
CFUs	Colony forming units
CP	Crude protein
DBWG	Daily body weight gain
EIOs	Edible inner organs
FCR	Feed conversion ratio
FI	Feed intake
GL	Gut length
HCL	Hydrochloric acid
IW	Initial weight
L*	Lightness
LAB	Lactic acid bacteria
MDA	Malondialdehyde
ME	Metabolic energy
MEA	Malt extract agar
MRS	De Man Rogosa Sharpe agar
NRC	National Research Council
SEM	Standard error of means
TBA	Thiobarbituric acid
VL	Villi length
WHC	Water-holding capacity
